# Loss of ten-eleven translocation 1 (TET1) expression as a diagnostic and prognostic biomarker of endometrial carcinoma

**DOI:** 10.1371/journal.pone.0259330

**Published:** 2021-11-03

**Authors:** Nien-Tzu Liu, Cherng-Lih Perng, Yu-Ching Chou, Pi-Shao Ko, Yi-Jia Lin, Yu-Chun Lin, Cheng-Chang Chang, Yu-Chi Wang, Hung-Sheng Shang, Tai-Kuang Chao

**Affiliations:** 1 Department of Pathology, Tri-Service General Hospital, National Defense Medical Center, Taipei, Taiwan; 2 Division of Clinical Pathology, Department of Pathology, Tri-Service General Hospital, National Defense Medical Center, Taipei, Taiwan; 3 School of Public Health, National Defense Medical Center, Taipei, Taiwan; 4 Department of Obstetrics and Gynecology, Tri-Service General Hospital, National Defense Medical Center, Taipei, Taiwan; Barts and The London School of Medicine and Dentistry Blizard Institute, UNITED KINGDOM

## Abstract

Endometrial carcinoma (EC) is the most common gynecological cancer. However, there is currently no routinely used biomarker for differential diagnosis of malignant and premalignant endometrial lesions. Ten-eleven translocation (TET) proteins, especially TET1, were found to play a significant role in DNA demethylation, via conversion of 5-methylcytosine (5-mC) to 5-hydroxymethylcytosine (5-hmC). TET1, 5-mC, and 5-hmC expression profiles in endometrial carcinogenesis are currently unclear. We conducted a hospital-based retrospective review of the immunohistochemical expression of TET1, 5-mC, and 5-hmC in 181 endometrial samples. A “high” TET1 and 5-hmC expression score was observed in all cases of normal endometrium (100.0% and 100.0%, respectively) and in most samples of endometrial hyperplasia without atypia (90.9% and 78.8%, respectively) and atypical hyperplasia (90.6% and 93.8%, respectively), but a “high” score was found in only less than half of the EC samples (48.8% and 46.5%, respectively). The TET1 and 5-hmC expression scores were significantly higher in normal endometrium and premalignant endometrial lesions than in ECs (p < 0.001). A “high” 5-mC expression score was observed more frequently for ECs (81.4%) than for normal endometrium (40.0%), endometrial hyperplasia without atypia (51.5%), and atypical hyperplasia (53.1%) (p < 0.001). We also found that TET1 mRNA expression was lower in ECs compared to normal tissues (p = 0.0037). TET1 immunohistochemistry (IHC) scores were highly proportional to the TET1 mRNA levels and we summarize that the TET1 IHC scoring can be used for biomarker determinations. Most importantly, a higher TET1 score in EC cases was associated with a good overall survival (OS) rate, with a hazard ratio (HR) of 0.31 for death (95% confidence interval: 0.11–0.84). Our findings suggest that TET1, 5-mC, and 5-hmC expression is a potential histopathology biomarker for the differential diagnosis of malignant and premalignant endometrial lesions. TET1 is also a potential prognostic marker for EC.

## Introduction

In the United States and most developed countries, EC is the most common gynecological malignancy, and its incidence is increasing. ECs have long been divided into two categories: type I and type II. The estrogen-dependent type I EC includes nuclear grade 1 (G1) and grade 2 (G2) endometrioid adenocarcinoma (EmAC). The less common, clinically aggressive estrogen-independent type II EC includes nuclear grade 3 (G3) EmAC, serous carcinoma (SC), and clear cell carcinoma (CC) [1–3]. Although most type I EmAC cases are diagnosed at an early stage, cases diagnosed at advanced stages usually present a poor survival rate. Hence, it is essential to identify EC at an early stage. However, there is no reliable biomarker that can be used to successfully distinguish between malignant and premalignant endometrial lesions [4]. Therefore, identification of biomarkers to improve the diagnosis is urgently required.

DNA methylation and demethylation play essential roles in modulating chromosome structure and regulating specific gene expression or repression during cell differentiation [5]. DNA methyltransferases are the main enzymes promoting DNA methylation [6]. Recent research has revealed extensive epigenetic modifications that are involved in endometrial carcinogenesis and this provides a window of opportunity for improved therapy [7,8]. Ten-eleven translocation enzymes (TET1, TET2, and TET3) constitute a family of dioxygenase, which can catalyze conversion of 5-mC to 5-hmC by oxidation, and they play a key role in DNA demethylation [9–12]. Altered expression of TET1 affects the balance between DNA methylation and demethylation and is associated with the onset and progression of several types of cancer. TET1 was first discovered in patients with a rare variant of t(10;11)(q22;q23) acute myeloid leukemia, and the gene is located on chromosome 10q21.3 [13]. TET1 is generally considered a tumor suppressor, while it is often down-regulated in multiple malignancies including breast, hepatic, pancreatic, gastric, and prostate cancers [5,14–17], but it has also been reported as a potential oncogene that contributes to aberrant hypomethylation in cancer [18]. Aberrant DNA methylation in EC has been widely studied in recent years [19], but the relationship between TET1, 5-mC, 5-hmC, and EC is still rarely reported [20]. The aim of this study was to investigate the TET1, 5-mC, and 5-hmC immunohistochemical expression patterns in different endometrial lesions in relation to clinicopathological characteristics of EC. To our knowledge, this study is the first to assess the usefulness of immunohistochemical evaluation of TET1, 5-mC, and 5-hmC as biomarkers for the differential diagnosis of normal endometrium, premalignant endometrial lesions, and EC. The prognostic significance of TET1 expression for OS was also established.

## Materials and methods

### Tissue microarray

Tissues from Chinese patients, embedded in paraffin wax, were retrospectively retrieved from the Pathology Department at Tri-Service General Hospital. All tissue microarray slides were prepared following the method according to the article by Hidalgo A in 2003 as the following steps. We used the 16G needle to punch paraffin wax cylinders (3 × 3 mm) on a paraffin wax block (2.5 × 2.5 cm). And then we used the 16G needle to obtain tissue cylinders, which were injected into the blank paraffin wax block. We used tissues derived from premalignant endometrial lesions and malignant ECs for tissue microarray construction. Once the tissue microarray was completed, we poured over a small amount of hot liquid paraffin wax over the tissue microarray surface and leveled the tissue cylinders with the block using a glass slide. The tissue microarray was incubated on the slide at 60° C for 15 minutes to blend together the paraffin wax from the blank block and the tissue cylinders. After incubation, the tissue microarray was chilled on ice and 5 μm sections were obtained using a rotatory microtome. Tissue microarray sections were mounted on sylanised slides and incubated overnight at 40°C [21]. We collected normal endometrium and endometrial hyperplasia (EH) specimens that obtained via biopsy and the main tumor part for EC in biopsies or hysterectomy samples. The diagnostic criteria was based on the 2020 classification of EH from the World Health Organization [22] and the 2018 International Federation of Gynecology and Obstetrics (FIGO) staging system classifications [23]. All the selected samples were obtained with written informed consent, and our study was approved by the Institutional Review Board of Tri-Service General Hospital (TSGHIRB No.:2-103-05-144 and 2-104-05-022). The tissue microarray consisted of 181 samples: 30 samples of normal endometrium, 33 of EH without atypia, 32 of atypical hyperplasia (AH), and 86 of EC, including 72 cases of EmAC, nine of SC, four of CC, and one case of mucinous carcinoma (MC). After tissue confirmation, all these cases were subjected to surgical hysterectomy. We classified the EC cases into type I tumors consisting of endometrioid histology, including grades 1 and 2, and type II tumors consisting of grade 3 endometrioid tumors or other nonendometrioid histology such as clear cell, mucinous, and serous for further evaluation.

### Immunohistochemistry staining

Immunohistochemistry was carried out obtained from the tissue microarray slides using anti-TET1 antibody (1:100; Abcam, Burlingame, CA, USA), anti-5mC antibody (1:1000; Abcam), and anti-5hmC antibody (1:100; Abcam) diluted in phosphate-buffered solution. The sections were incubated with horseradish peroxidase-labeled antibody (Dako, Carpinteria, CA, USA) for 1 hour at room temperature, and peroxidase activity was visualized using a chromogenic solution of diaminobenzidine at room temperature. The cases were assessed by two gynecological pathologists in a double-blind manner. The immunoreactivity was graded arbitrarily and semiquantitatively by considering the intensity and percentage of staining on the tissue microarray slides as described previously [23]. The intensity of 5-hmC, 5-mC, and TET1 staining in individual tumor cells was scored as 0 (no staining), 1+ (weak intensity), 2+ (moderate intensity), or 3+ (strong intensity). The percentage of cells for each intensity was estimated from 0 to 100. For semiquantitative analysis of these three markers’ expression, the absolute score was calculated by multiplying the estimated percentage of stained cells at each intensity by the corresponding intensity value, which produced an immunostaining score from 0 to 300. The scoring of expression profiles for TET1, 5-hmC, and 5-mC was performed by two gynecopatholgists respectively. We evaluate the average score of each sample in three different positions. To compare absolute scores of these three markers between different endometrial lesions, the optimal cut-off value was determined using a receiver-operating characteristic (ROC) curve. TET1^low^ was defined as a score ≤ 20, and TET1^high^ was defined as a score >20; 5-hmC^low^ was defined as a score ≤80, and 5-hmC^high^ was defined as a score >80; whereas 5-mC^low^ was defined as a score ≤270, and 5-mC^high^ was defined as a score >270.

### RNA isolation and TET1 quantitative real-time RT-PCR

Total RNA was extracted from samples of 15 normal endometrium and 15 EC tissues using Presto FFPE RNA Mini Kit (Geneaid, New Taipei City, Taiwan). Cut up 2~5 sections of 5~20 μm thick FFPE sections for RNA extraction and finally eluted with 50 μL of RNase-free Water. The TET1 mRNA expression level was measured by real time one-step RT-PCR normalized by GAPDH house-keeping gene. The TET1 and GAPDH RT-PCR primers were followed by previous design [24]. The one-step RT-PCR amplification was performed at 50°C for 10 mins and 95°C for 2 mins followed by 40 cycles at 95°C for 3 s, 55°C for 15 s, and 72°C for 5 s using a Roche LightCycler 2.0 Real-Time PCR System (Roche Ltd., Basel, Switzerland) with 5 μL of RNA and 20 μL of total volume with SensiFAST SYBR No-ROX One-Step Kit (BIOLINE, London, UK).

### Statistical analysis

Patients’ clinical data were retrieved from hospital patient files. All values are expressed as mean ± standard error of mean and as percentages. ROC curve analysis is applied to measure the diagnostic accuracy. We choose the maximization of the sum of sensitivity and specificity as the cut-off point. Therefore, the cut-off point, sensitivity, specificity, and area under the curve (AUC) of TET1 are 20, 0.512, 0.937, and 0.724, respectively; 5-hmC is 80, 0.535, 0.905, and 0.720; whereas 5-mC is 270, 0.814, 0.512, and 0.665 (S1 Fig). Comparisons of the expression levels of TET1, 5-hmC, and 5-mC between different groups of normal endometrial, EH, AH, and EC samples were analyzed using analysis of variance and chi-squared tests. A chi-squared test or Fisher’s exact test was used to identify associations between expression levels of these three markers and clinicopathological characteristics.

Data were assessed using Cox regression analysis. Kaplan–Meier survival curves were compared using a log-rank test. A two-sided p < 0.05 was considered significant. All analyses were performed using IBM SPSS Statistics for Windows (version 21; IBM Corp, Armonk, NY, USA).

## Results

### TMA evaluation

**Fig 1:**
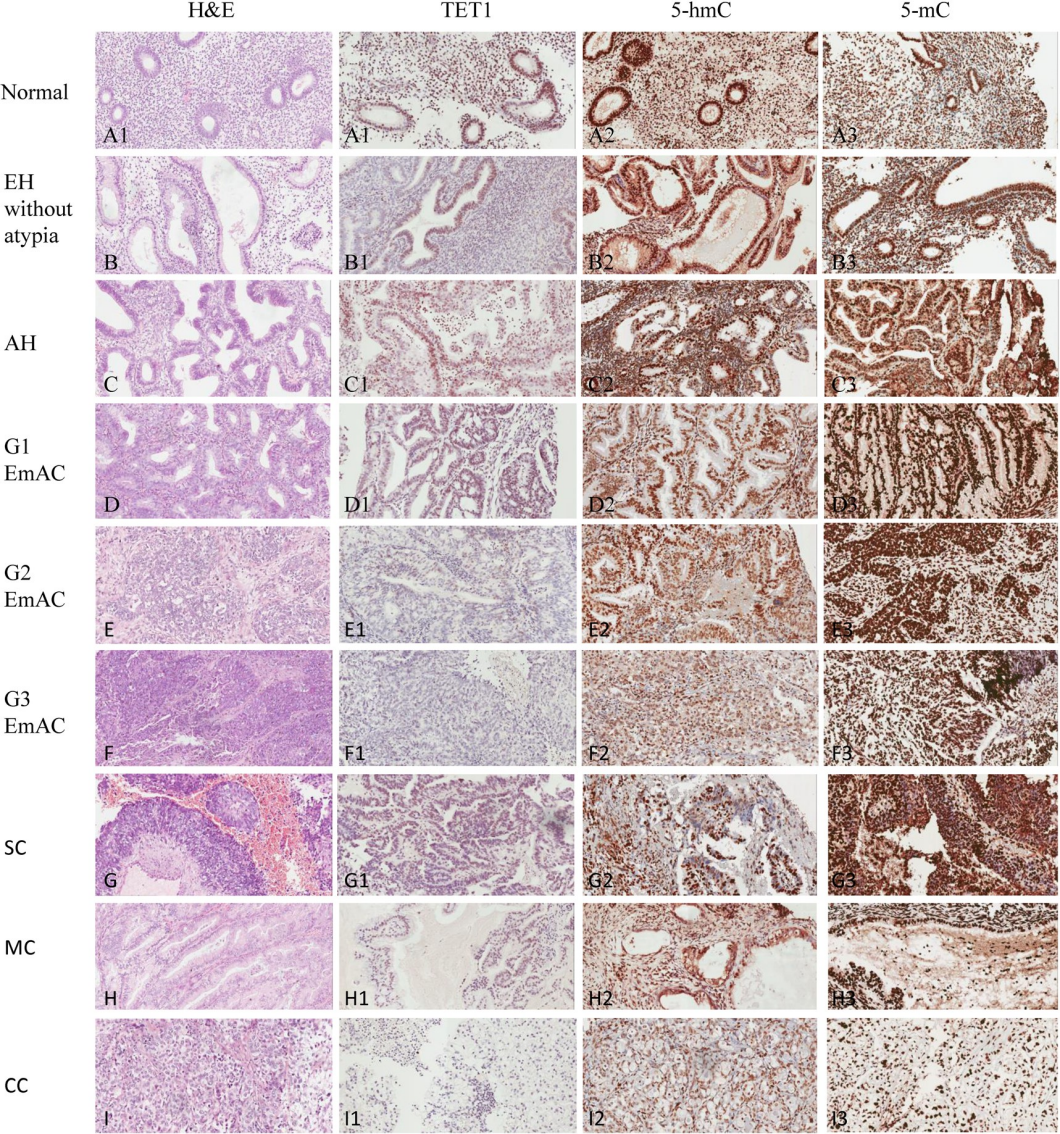
H&E staining in samples of normal endometrium (A), premalignant endometrial lesions, including EH without atypia (B) and AH (C), as well as malignant samples of EC, including G1 of EmAC (D), G2 of EmAC (E), G3 of EmAC (F), SC (G), MC (H), and CC (I); *TET1* expression in samples of normal endometrium (A1), EH without atypia (B1), AH (C1), G1 of EmAC (D1), G2 of EmAC (E1), G3 of EmAC (F1), SC (G1), MC (H1), and CC (I1); *5-hmC* expression in samples of normal endometrium (A2), EH without atypia (B2), AH (C2), G1 of EmAC (D2), G2 of EmAC (E2), G3 of EmAC (F2), SC (G2), MC (H2), and CC (I2); *5-mC* expression in samples of normal endometrium (A3), EH without atypia (B3), AH (C3), G1 of EmAC (D3), G2 of EmAC (E3), G3 of EmAC (F3), SC (G3), MC (H3), and CC (I3).

To evaluate the expression levels of TET1, 5-hmC, and 5-mC in normal endometrium and endometrial lesions, including EH without atypia, AH, and EC, we performed IHC analysis. TET1, 5-hmC, and 5-mC show mainly intranuclear expression. We found a significant variation in levels of these three markers between the samples of premalignant endometrial lesions and malignant lesions. Fig 1 shows hematoxylin & eosin staining, TET1, 5-hmC and 5-mC in samples of normal endometrium, premalignant endometrial lesions, including EH without atypia and AH, and malignant samples of EC. Generally, high intranuclear immunoreactivity scores were observed for TET1 in all samples of normal endometrium (30/30, 100%) and most samples of premalignant endometrial lesions, including EH without atypia (30/33, 90.9%) and AH (29/32, 90.6%); whereas significantly lower TET1 expression was observed in EC cases (42/86, 48.8%), including EmAC, SC, CC, and MC (p < 0.001) (Table 1). Moreover, 5-hmC immunostaining scores were also high in all samples of normal endometrium (30/30, 100%), most samples of EH without atypia (26/33, 78.8%), and nearly all samples of AH (30/32, 93.8%), but they were high only in less than half of the EC samples (40/86, 46.5%) (p < 0.001) (Table 1). A similar relationship was noted for 5-hmC expression. The 5-mC immunostaining score was high in most samples of EC (70/86, 81.4%), but low in samples of normal endometrium (12/30, 40.0%), EH without atypia (17/33, 51.5%), and AH (17/32, 53.1%) (Table 1). In contrast, 5-mC expression showed the opposite trend, with higher immunostaining scores in EC samples, compared with low immunostaining scores in samples of normal endometrium and premalignant endometrial lesions (p < 0.001). There was wide variation in both the extent and intensity of TET1 staining between premalignant endometrial lesions and neoplastic endometrial samples; differential expression of 5-mC and 5-hmC was also observed. The difference in the TET1, 5-mC, and 5-hmC scores between the premalignant and malignant endometrial lesion groups was significant (p < 0.001).

**Table 1:** Chi-squared test for TET1, 5-hmC, and 5-mC expression scores based on the area of staining and the intensity of color reaction.

	Normal n (%)	EH n (%)	AH n (%)	EC n (%)	p-value
TET1, n	30	33	32	86	< 0.001
≤20	0 (0.0)	3 (9.1)	3 (9.4)	44 (51.2)	
>20	30 (100.0)	30 (90.9)	29 (90.6)	42 (48.8)	
5-hmC, n	30	33	32	86	< 0.001
≤80	0 (0.0)	7 (21.2)	2 (6.3)	46 (53.4)	
>80	30 (100.0)	26 (78.8)	30 (93.8)	40 (46.5)	
5-mC, n	30	33	32	86	< 0.001
≤270	18 (60.0)	16 (48.5)	15 (46.9)	16 (18.6)	
>270	12 (40.0)	17 (51.5)	17 (53.1)	70 (81.4)	

AH, atypical hyperplasia; EC, endometrial carcinoma; EH, endometrial hyperplasia without atypia; Normal, normal endometrium.

**Fig 2:**
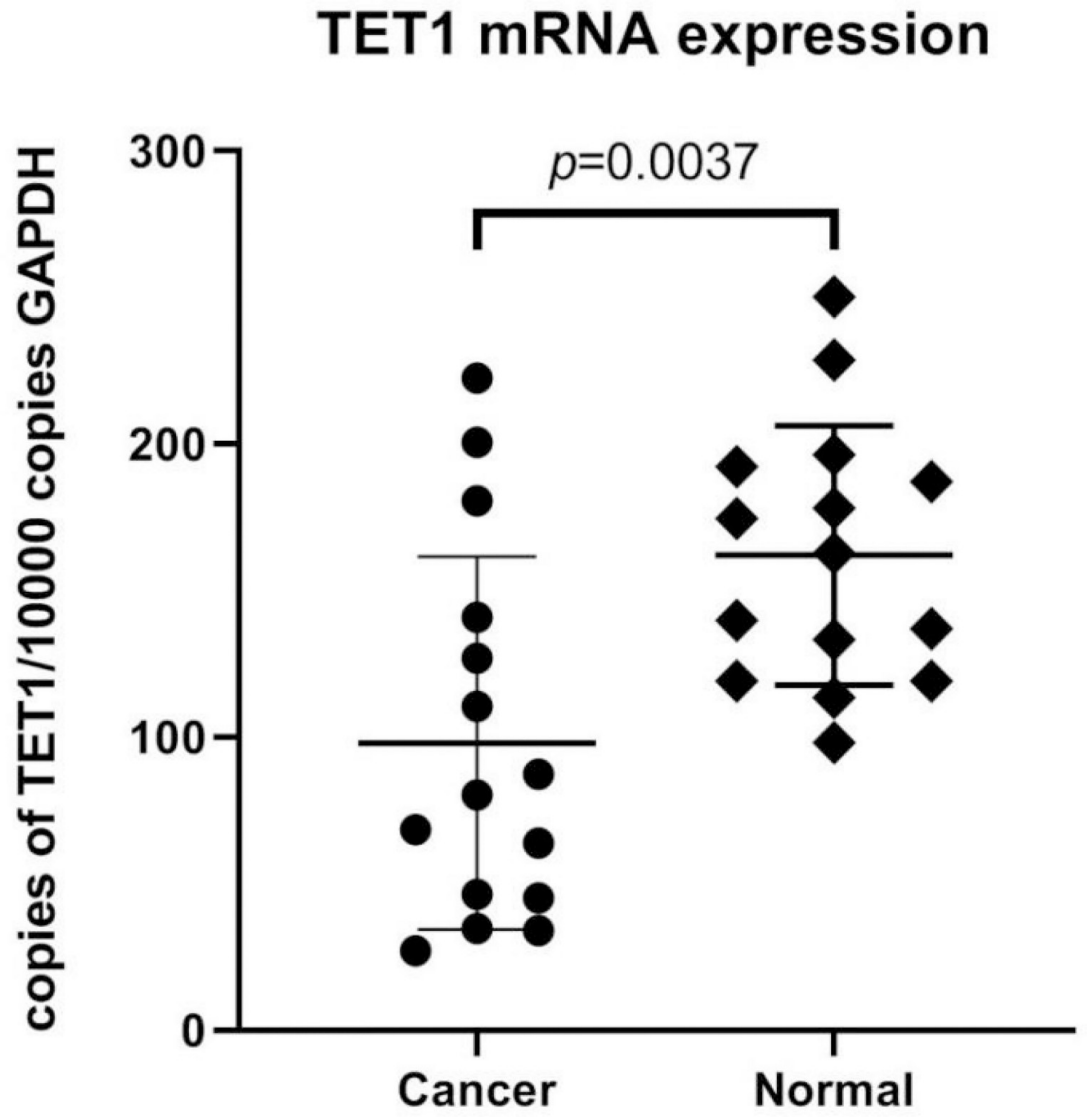
TET1 mRNA expression in normal endometrium and ECs.

**Fig 3:**
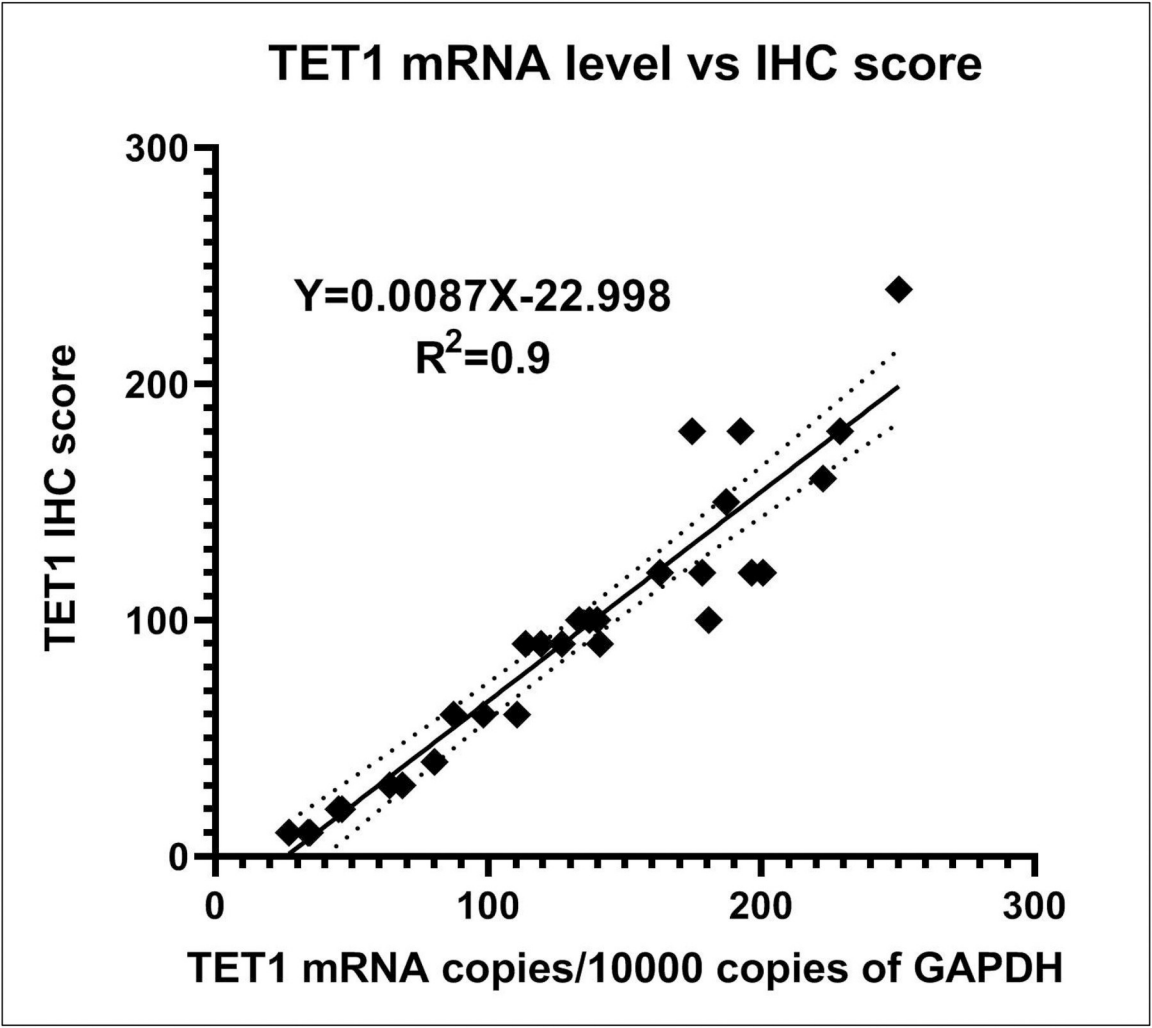
Comparison of TET1 IHC score and TET1 mRNA level. The two parameters were compared and analyzed by simple linear regression. The TET1 mRNA copies were calculated and shown with every 10,000 copies of GAPDH. The dotted line means the 95% confidence interval of simple linear regression analysis.

For further supporting the IHC results, we performed RT-PCR of TET1 to quantitatively confirm differential expression profiles at the mRNA level. The TET1 mRNA expression level was low in ECs compared with those in normal endometrium (p = 0.0037), as shown in Fig 2. The comparison of TET1 IHC scores and TET1 mRNA levels was shown in Fig 3. The R^2^ of simple linear regression with these two parameters was 0.9. Therefore, the TET1 IHC scores were highly proportional to the TET1 mRNA levels referenced to the GAPDH mRNA. The TET1 IHC scoring can be used for biomarker level determinations. The real time RT-PCR results of TET1 and GAPDH genes were shown in Table 2.

**Table 2:** The real time RT-PCR results of TET1 and GAPDH genes.

Tissue samples	TET1 Ct	GAPDH Ct	GAPDH/TET1 ΔCt	copies TET1/10000 copies GAPDH 10000*(2^-ΔCt)	TET1 IHC score
EC-01	28.18	22.69	5.49	222.51	160
EC-02	32.94	25.15	7.79	45.18	20
EC-03	28.04	21.89	6.15	140.82	90
EC-04	28.63	21.34	7.29	63.90	30
EC-05	25.38	19.74	5.64	200.54	120
EC-06	25.35	18.85	6.5	110.49	60
EC-07	25.71	19.92	5.79	180.73	100
EC-08	24.62	18.32	6.3	126.91	90
EC-09	30.25	23.29	6.96	80.32	40
EC-10	28.59	20.84	7.75	46.45	20
EC-11	31.69	24.85	6.84	87.29	60
EC-12	32.85	24.32	8.53	27.05	10
EC-13	32.27	24.07	8.2	34.01	10
EC-14	29.1	21.91	7.19	68.48	30
EC-15	29.19	21.02	8.17	34.72	10
Normal-01	26.69	20.99	5.7	192.37	180
Normal-02	26.98	21.24	5.74	187.11	150
Normal-03	34.98	28.59	6.39	119.24	90
Normal-04	32.21	25.54	6.67	98.20	60
Normal-05	28.61	22.22	6.39	119.24	90
Normal-06	25.11	19.79	5.32	250.33	240
Normal-07	26.84	20.9	5.94	162.89	120
Normal-08	27.3	20.84	6.46	113.59	90
Normal-09	26.01	19.82	6.19	136.97	100
Normal-10	26.74	20.93	5.81	178.24	120
Normal-11	26.16	20	6.16	139.85	100
Normal-12	27.96	22.12	5.84	174.58	180
Normal-13	26.24	20.57	5.67	196.41	120
Normal-14	26.86	21.41	5.45	228.76	180
Normal-15	28.93	22.7	6.23	133.22	100

### Clinicopathological correlation

We also analyzed the association between the clinicopathologic features and these three markers in ECs, including CC, EmAc, MC, and SC (TET1 in Table 3; 5-hmC in Table 4; 5-mC in Table 5). There were no significant associations between TET1, 5-hmC, and 5-mC scores and age (p = 0.814, 0.459, 0.226, respectively), FIGO stage (p = 0.966, 1.000, 0.750, respectively), or subtype of EC (p = 0.902, 0.509, 0.225, respectively, for the various histological type comparisons and p = 0.806, 0.971, 1.000, respectively, for type I and type II EC comparisons). Although a high 5-mC expression score was noted for high nuclear grade (p = 0.002), no significant associations between TET1 and 5-hmC scores and nuclear grade were observed (p = 0.213 and 0.895, respectively).

**Table 3:** Association of TET1 expression score with clinicopathological features in EC.

	TET1 ^low^	TET1 ^high^	
Characteristic	≤20	>20	p-value
Patients (n)	44	42	
Age (years)			0.814
Range	31–83	34–88	
Mean ± SEM	55.13 ± 1.62	55.72 ± 1.87	
FIGO stage (n [%])			0.966
I, II	30 (50.0)	30 (50.0)	
III, IV	14 (53.8)	12 (46.2)	
Nuclear grade (n [%])			0.213
G1	18 (46.2)	21 (53.8)	
G2	14 (60.9)	9 (39.1)	
G3	12 (50.0)	12 (50.0)	
Histological type (n [%])			0.902^a^
CC	2 (50.0)	2 (50.0)	
EmAC	37 (51.4)	35 (48.6)	
MC	0 (0.0)	1 (100.0)	
SC	5 (55.6)	4 (44.4)	
Histological type (n [%])			0.806
Type I EC (EmAC G1 and G2)	32 (51.6)	30 (48.4)	
Type II EC (EmAC G3, MC, CC, and SC)	12 (50.0)	12 (50.0)	

^a^ Fisher’s exact test. CC, clear cell carcinoma; EmAC, endometrioid adenocarcinoma; MC, mucinous carcinoma; SC, serous carcinoma; SEM, standard error of mean.

**Table 4:** Association of 5-hmC expression score with clinicopathological features in EC.

	5-hmC ^low^	5-hmC ^high^	
Characteristic	≤80	>80	p-value
Patients (n)	46	40	
Age (years)			0.459
Range	31–79	33–88	
Mean ± SEM	54.55 ± 1.64	56.37 ± 1.83	
FIGO stage (n [%])			1.000
I, II	32 (53.3)	28 (46.7)	
III, IV	14 (53.8)	12 (46.2)	
Nuclear grade (n [%])			0.895
G1	20 (51.3)	19 (48.7)	
G2	13 (56.5)	10 (43.5)	
G3	13 (54.2)	11 (45.8)	
Histological type (n [%])			0.509^a^
CC	3 (75.0)	1 (25.0)	
EmAC	37 (51.4)	35 (48.6)	
MC	0 (0.0)	1 (100.0)	
SC	6 (66.7)	3 (33.3)	
Histological type (n [%])			0.971
Type I EC (EmAC G1 and G2)	34 (54.8)	28 (45.2)	
Type II EC (EmAC G3, MC, CC, and SC)	12 (50.0)	12 (50.0)	

^a^ Fisher’s exact test. CC, clear cell carcinoma; EmAC, endometrioid adenocarcinoma; MC, mucinous carcinoma; SC, serous carcinoma; SEM, standard error of mean.

**Table 5:** Association of 5-mC expression score with clinicopathological features in EC.

	5-mC ^low^	5-mC ^high^	
Characteristic	≤270	>270	p-value
Patients (n)	16	70	
Age (years)			0.226
Range	31–75	33–88	
Mean ± SEM	52.47 ± 2.61	56.27 ± 1.37	
FIGO stage (n [%])			0.750^a^
I, II	10 (16.7)	50 (83.3)	
III, IV	6 (23.1)	20 (76.9)	
Nuclear grade (n [%])			0.002^a^
G1	12 (30.8)	27 (69.2)	
G2	0 (0.0)	23 (100.0)	
G3	4 (16.7)	20 (83.3)	
Histological type (n [%])			0.225^a^
CC	0 (0.0)	4 (100.0)	
EmAC	13 (18.1)	59 (81.9)	
MC	1 (100.0)	0 (0.0)	
SC	2 (22.2)	7 (77.8)	
Histological type (n [%])			1.000^a^
Type I EC (EmAC G1 and G2)	12 (19.4)	50 (80.6)	
Type II EC (EmAC G3, MC, CC, and SC)	4 (16.7)	20 (83.3)	

^a^ Fisher’s exact test. CC, clear cell carcinoma; EmAC, endometrioid adenocarcinoma; MC, mucinous carcinoma; SC, serous carcinoma; SEM, standard error of mean.

### Survival analyses

**Fig 4:**
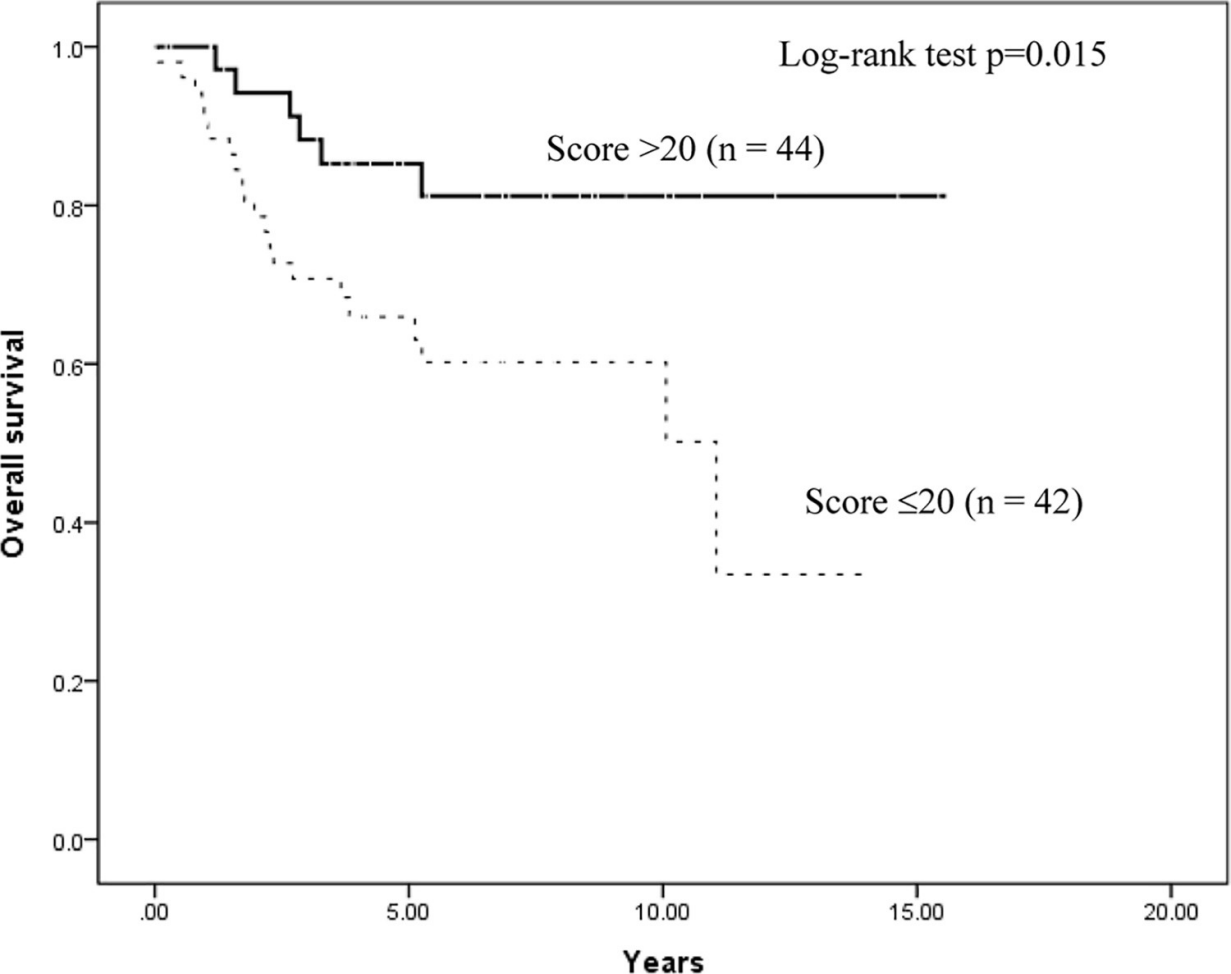
Kaplan–Meier analysis stratified according to TET1 expression score. Analysis to assess the prognostic value of the TET1 immunoreactivity in relation to OS.

**Fig 5:**
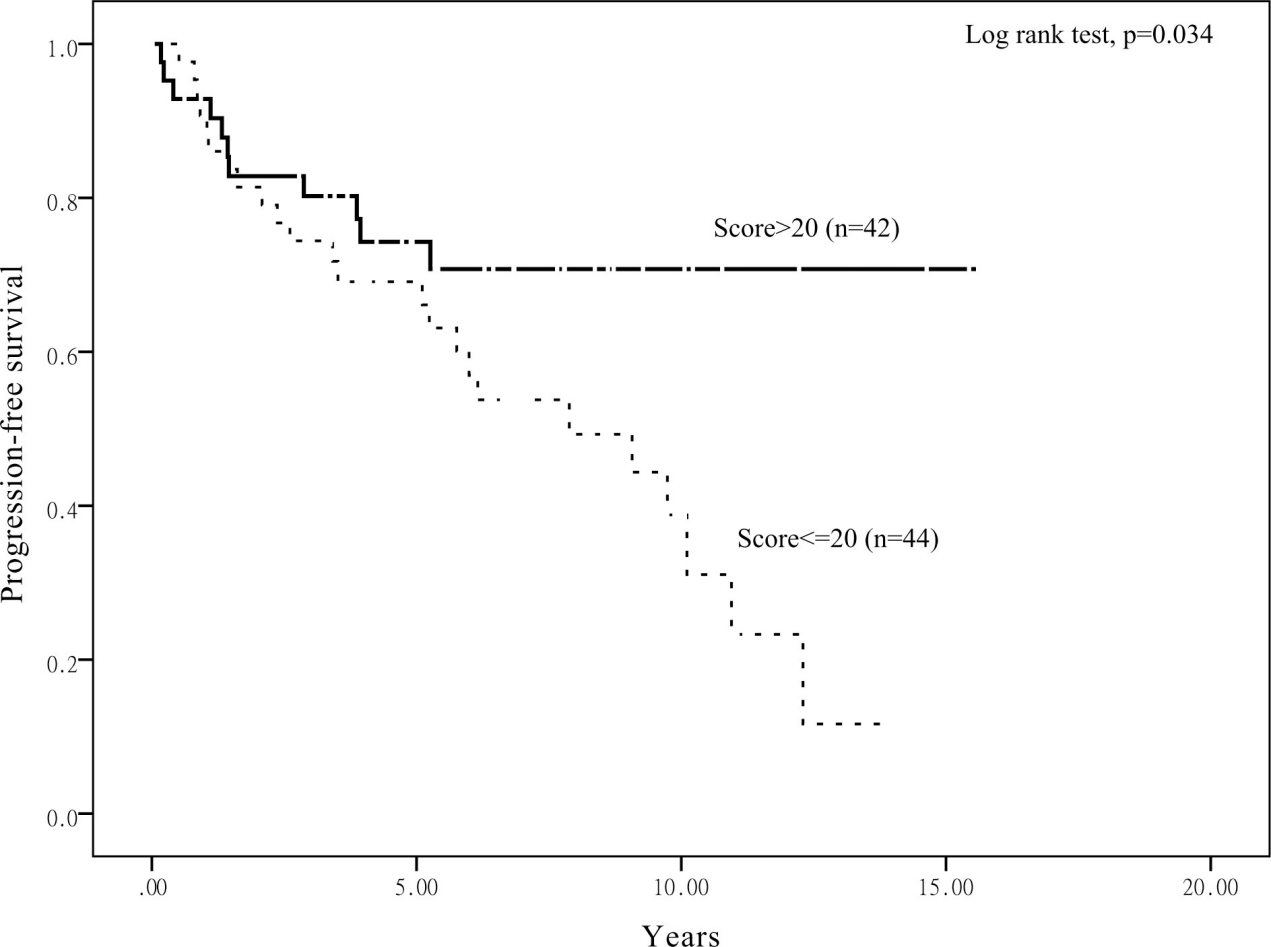
Kaplan–Meier analysis stratified according to TET1 expression score. Analysis to assess the prognostic value of the TET1 immunoreactivity in relation to PFS.

Kaplan–Meier survival analysis stratified according to TET1 expression score to assess the prognostic value of the TET1 immunoreactivity in relation to OS is shown in Fig 4. Patients with EC with a lower score (≤20) had worse OS than those with a higher score (>20) (p = 0.015). Progression-free survival (PFS) also had consistent results (p = 0.034), as shown in Fig 5. Furthermore, we also analyzed OS and PFS according to 5-hmC and 5-mC expression scores, but the results had not significantly different (S2–S5 Figs).

Univariate survival analysis (Table 6) revealed that higher TET1 expression level (>20) resulted in a HR of death = 0.34 (95% confidence interval [CI]: 0.14–0.85; p < 0.05), higher FIGO stage resulted in an HR of death = 8.41 (95% CI: 3.28–21.58; p < 0.01), higher nuclear grade resulted in an HR of death = 3.26 (95% CI: 1.38–7.74; p < 0.01), and type II EC (including G3 of EmAC, MC, CC and SC) resulted in an HR of death = 3.52 (95% CI: 1.62–7.63; p < 0.01). After adjusting for age, FIGO stage, and histological type, the Cox proportional hazards regression analysis indicated that TET1^high^ score (>20) is an independent predictor of OS, with higher TET1 expression levels conferring an HR of death = 0.31 (95% CI: 0.11–0.84; p < 0.05).

**Table 6:** Multivariate survival analysis of clinicopathological factors in 86 patients with EC.

Variable	Univariate analysis crude HR (95% CI)	Multivariate adjusted HR (95% CI)
Age (years)	1.05 (1.02–1.08)**	1.05 (1.02–1.10)**
TET1 expression^a^		
Low	1.00 (Ref.)	1.00 (Ref.)
High	0.34 (0.14–0.85)*	0.31 (0.11–0.84)*
5-hmC expression^b^		
Low	1.00 (Ref.)	
High	0.80 (0.37–1.73)	–
5-mC expression^c^		
Low	1.00 (Ref.)	
High	1.98 (0.59–6.58)	–
FIGO stage		
I, II	1.00 (Ref.)	1.00 (Ref.)
III, IV	8.41 (3.28–21.58)**	7.70 (2.72–21.76) **
Nuclear grade		
G1	1.00 (Ref.)	
G2	0.82 (0.27–2.44)	–
G3	3.26 (1.38–7.74)**	–
Histological type		
Type I EC (EmAC G1 and G2)	1.00 (Ref.)	1.00 (Ref.)
Type II EC (EmAC G3, MC, CC, and SC)	3.52 (1.62–7.63)**	3.46 (1.37–8.74)**

^a^ Low expression of TET1 is represented as ≤20; high expression of TET1 is represented as >20.

^b^ Low expression of 5-hmC is represented as ≤80; high expression of 5-hmC is represented as >80.

^c^ Low expression of 5-mC is represented as ≤270; high expression of 5-mC is represented as >270.

* p < 0.05

** p < 0.01. CC, clear cell carcinoma; CI, confidence interval; EC, endometrial carcinoma; EmAC, endometrioid adenocarcinoma; HR, hazard ratio; MC, mucinous carcinoma; Ref., reference group; SC, serous carcinoma.

## Discussion

EC is a multifactorial disease, and the molecular mechanism underlying its development and progression is poorly understood. The increasing incidence of EC worldwide requires the development of accurate biomarkers to distinguish premalignant and malignant endometrial lesions. Molecular diagnostic tools have been suggested as novel methods to improve the differential diagnosis of endometrial premalignant and malignant lesions. Recently, research has been focused on elucidating the molecular and genetic characteristics of EC to provide new insights into its biology, which can enable development of innovative treatments [7]. Several genetic alterations have been observed in patients with EC, including microsatellite instability, mutations of PTEN, K-RAS, and β-catenin genes, as well as epigenetic changes, such as aberrant DNA methylation and histone modifications, which play a significant role in cancer development [25–27].

For the reason that several diagnostic markers, such as phosphatase and tensin homolog (PTEN), have been proposed. PTEN, a tumor suppressor gene controlling the PI3K/AKT pathway by phosphorylating PIP3 at the cell membrane, is the most common genetic alteration (30–80%) in endometrial carcinomas. A system review and meta-analysis down by Antonio Raffone, revealed that immunohistochemistry for PTEN showing low diagnostic usefulness in the differential diagnosis between benign and premalignant endometrial hyperplasia. [28]. Another well-known transcriptional factor is Sex-determining region Y-box 2 (SOX2), which is an essential in the self-renewal and pluripotency of embryonic stem cells, affecting in cell survival and progression of cancers. As the results shown in Cancer Science by Kaoru Yamawaki in 2017, SOX2 expression is correlated with histological grade and poor prognosis in endometrial cancer. Furthermore, high SOX2 accompanied by low p21 expressions in the patients of advanced endometrial cancer were associated with the most unfavorable outcomes, which indicated that the expression of SOX2 and p21 may be a useful biomarker for disease prognosis in endometrial cancer patients [29].

It is important to distinguish between AH and EC because such a distinction provides a basis for the clinician to evaluate the risks of conservative progestin‐based therapy rather than surgical therapy in selected younger patients who wish to retain fertility [30]. The histologic evaluation, such as cytological atypia, is regarded as the gold standard in classify malignant carcinomas, premalignant and benign endometrial lesions. However, histological classification may be affected by some issues, such as low reproducibility and ambiguous features [31]. Cytological features alone would be of little help in distinguishing between AH and well-differentiated EmAC [32]. For approximately 15–50% of EC cases of permanent hysterectomy specimens, the initial biopsy or curettage diagnosis indicated AH [25].

DNA methylation at the C-5 position of cytosines represents an important epigenetic modification involved in tissue differentiation and is involved in many diseases, especially cancers [33]. A specific DNA methylation pattern is determined not only by the attachment of methyl group to cytosine but also as a result of the process of DNA demethylation [34,35]. To improve reliability of the differential diagnosis, we evaluated whether DNA demethylation could be used as a predictive factor to confirm the diagnosis. TET family proteins can catalyze the conversion of 5-mC in the DNA to 5-hmC, contributing to DNA demethylation [36]. TET1 initiates the process of DNA demethylation, which often plays a role in tumor suppression in cancers, to promote DNA hydroxymethylation in the promoter regions of its target genes. Thus, metabolic disorder due to mutations in metabolic genes, such as isocitrate dehydrogenase, fumarate hydratase, and succinate dehydrogenase, could reduce TET enzymatic activity, leading to the impairment of DNA hydroxymethylation, and could contribute to cancer progression [37,38]. To date, several studies have confirmed the important role of TET proteins in DNA demethylation and their relationship with changes in DNA methylation patterns in tumors [20]. Decreased 5-hmC levels were found in many solid tumors (including glioma, melanoma, gastric, breast, colon, liver, lung, prostate, and pancreatic cancers) [16,33,39–47]. Recently, Yang et al. showed that TET1 functions as a tumor suppressor in EC. In addition, TET1 overexpression enhances 5-hmC levels in the EC cells [37]. In this study, we found that the expression of TET1 and 5-hmC is higher in normal and premalignant endometrial lesions and decreased on progression to EC (p < 0.001). Moreover, 5-mC expression showed the opposite trend. Higher expression of 5-mC was observed in EC samples than in normal and premalignant endometrial lesions (p < 0.001). This finding indicates that TET1, 5-hmC, and 5-mC play an important role in the progression of premalignant endometrial lesions to EC.

Clinical parameters showed that patients with EC with low TET1 expression have shorter OS than those with high expression of TET1. A previous report suggested that low expression levels of TET1 messenger RNA predict a poor OS rate for patients with EC [20]. Our findings revealed that a reduction in the TET1 score was associated independently and significantly with poor OS in patients with EC, which is similar to previous research findings. To our knowledge, no precise data analysis for TET1, 5-hmC, and 5-mC expression levels associated with clinicopathological factors, including age, tumor FIGO stage, histological type, and nuclear grade, is available for patients with EC. Hence, our study aimed at evaluating the relationship between these factors. The multivariate survival analysis revealed that high TET1 expression resulted in an HR of death = 0.31 (95% CI: 0.11–0.84; p < 0.05), and it is also statistically significant in relation to FIGO stage (p < 0.01) and histologic type (p < 0.01) in patients with EC.

Although previously published research article has reported that TET1 mRNA expression and correlated 5-hmC levels are reduced in endometrial cancer tissues [48]. Compared to its focus on PCR analysis for mRNA expression, our study used immunohistochemical method for expression profiles. The immunohistochemical examination is much a rapider and cheaper way for pathologists while clinically practicing in differential diagnosis of malignant and premalignant endometrial lesions and survival prediction. Our data also revealed that TET1 IHC scores were highly proportional to the TET1 mRNA levels. Therefore, we summarize that the TET1 IHC scoring can be used for biomarker level determinations.

In summary, we found that the expression profiles of TET1, 5-hmC, and 5-mC in ECs correlate with several factors, including tumor FIGO stage, tumor histological type, and nuclear grade. We also determined that TET1, known to suppress various cancers, might be a useful biomarker for differential diagnosis of malignant and premalignant endometrial lesions, and it appears to be a significant prognostic factor for patients with EC. Considering that this study is limited by the small number of samples, it may not be appropriate to apply its findings to the whole population directly. Further, larger population-based research is suggested for the validation of TET1 as a new biomarker for distinguishing normal endometrium, EH, AH, and EC in pathological diagnosis.

## Supporting information

S1 Fig Receiver-operating characteristic (ROC) curve. ROC curve analysis of TET1 (continuous line), 5-hmC (dotted line), and 5-mC (dashed line), respectively.
(TIF)

S2 Fig Kaplan–Meier analysis stratified according to 5-hmC expression score. Analysis to assess the prognostic value of the 5-hmC immunoreactivity in relation to OS.
(TIF)

S3 Fig Kaplan–Meier analysis stratified according to 5-hmC expression score. Analysis to assess the prognostic value of the 5-hmC immunoreactivity in relation to PFS.
(TIF)

S4 Fig Kaplan–Meier analysis stratified according to 5-mC expression score. Analysis to assess the prognostic value of the 5-mC immunoreactivity in relation to OS.
(TIF)

S5 Fig Kaplan–Meier analysis stratified according to 5-mC expression score. Analysis to assess the prognostic value of the 5-mC immunoreactivity in relation to PFS.
(TIF)
