# Supplementary figures and images for: Loss of ten-eleven translocation 1 (TET1) expression as a diagnostic and prognostic biomarker of endometrial carcinoma
Source: PLoS One. 2021 Nov 3;16(11):e0259330. doi: 10.1371/journal.pone.0259330 (PMC8565757; doi:10.1371/journal.pone.0259330)

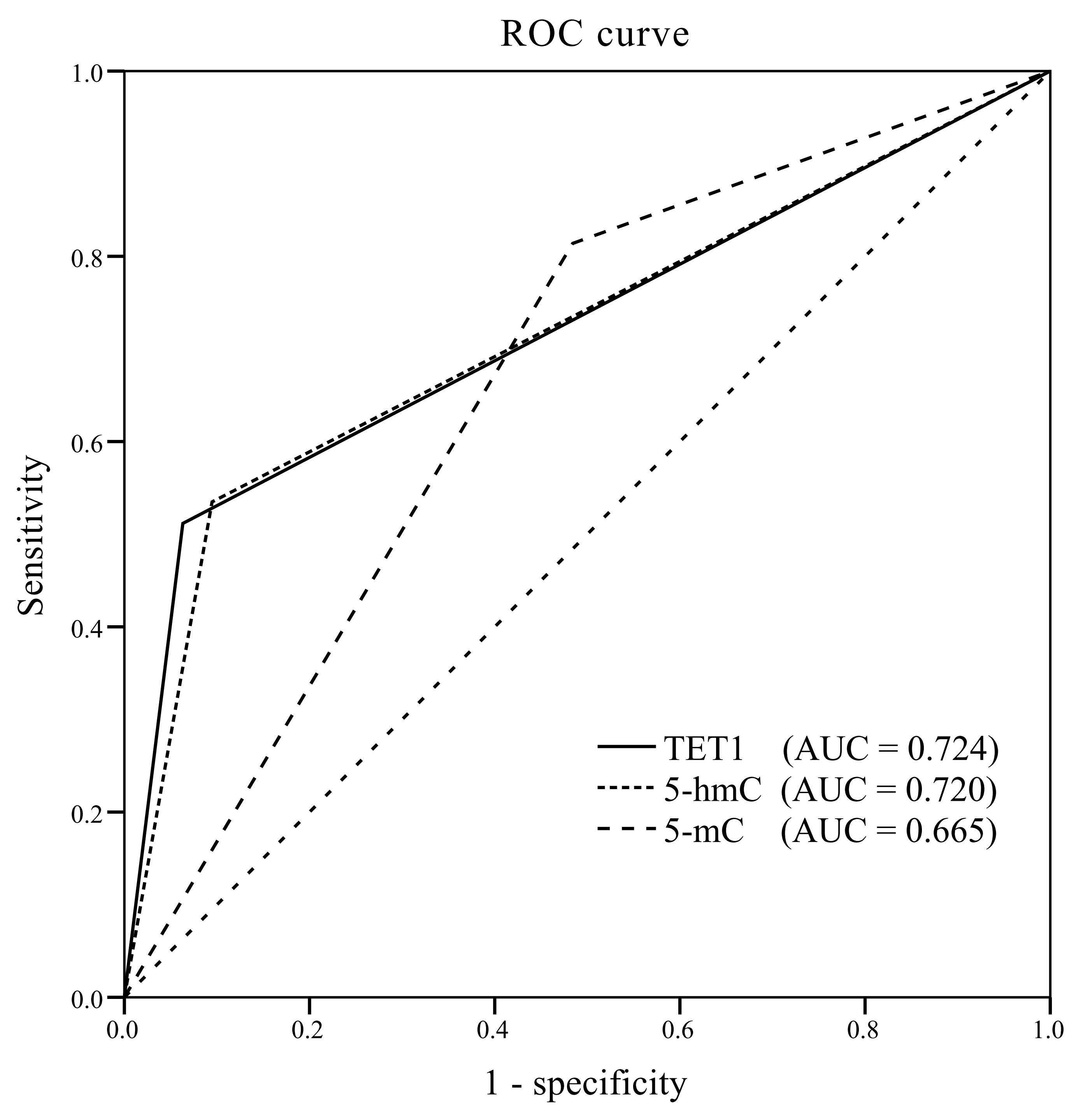

Supplement: S1 Fig — ROC curve analysis of TET1 (continuous line), 5-hmC (dotted line), and 5-mC (dashed line), respectively. (TIF) [file pone.0259330.s001.tif]

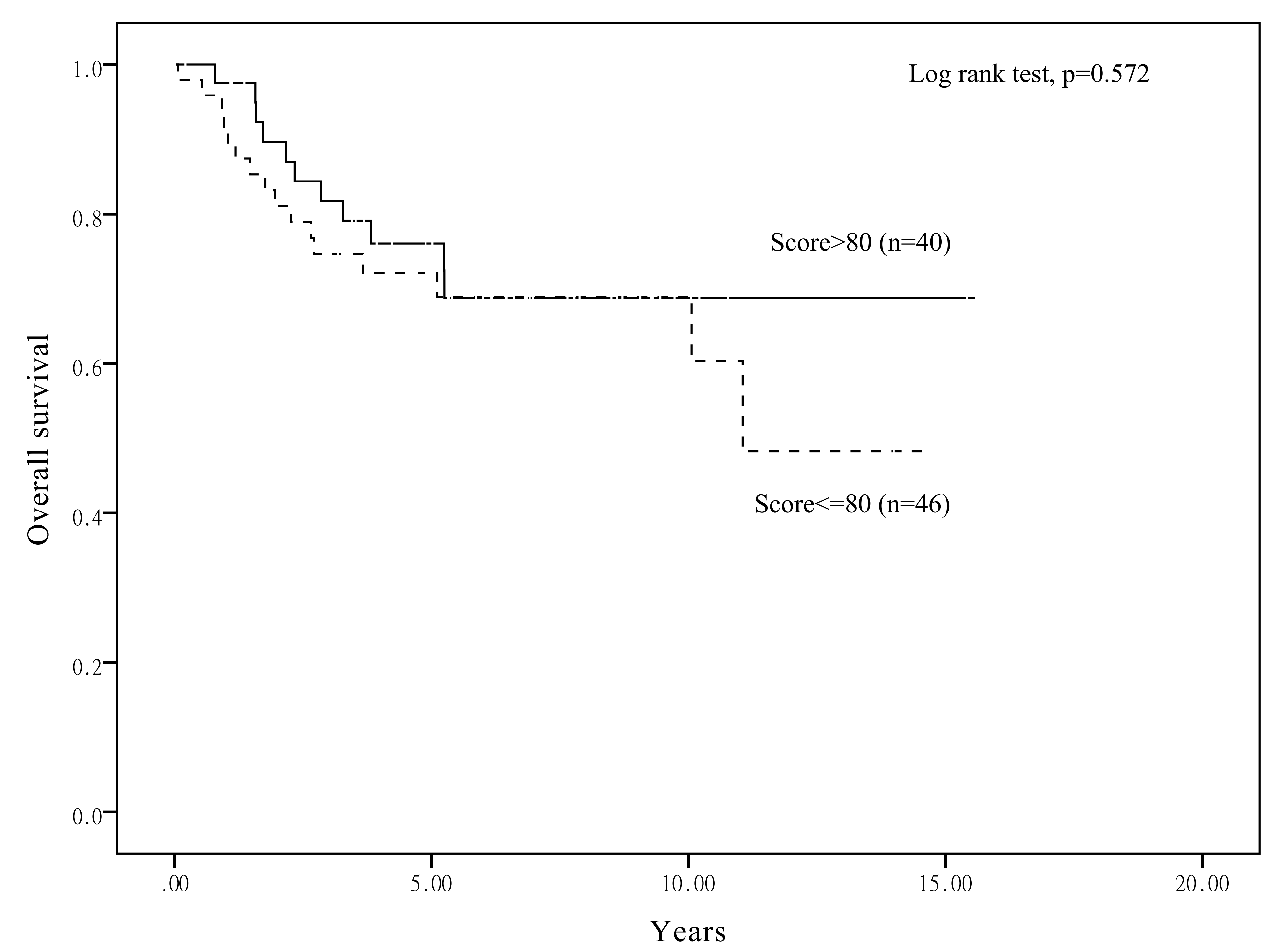

Supplement: S2 Fig — Analysis to assess the prognostic value of the 5-hmC immunoreactivity in relation to OS. (TIF) [file pone.0259330.s002.tif]

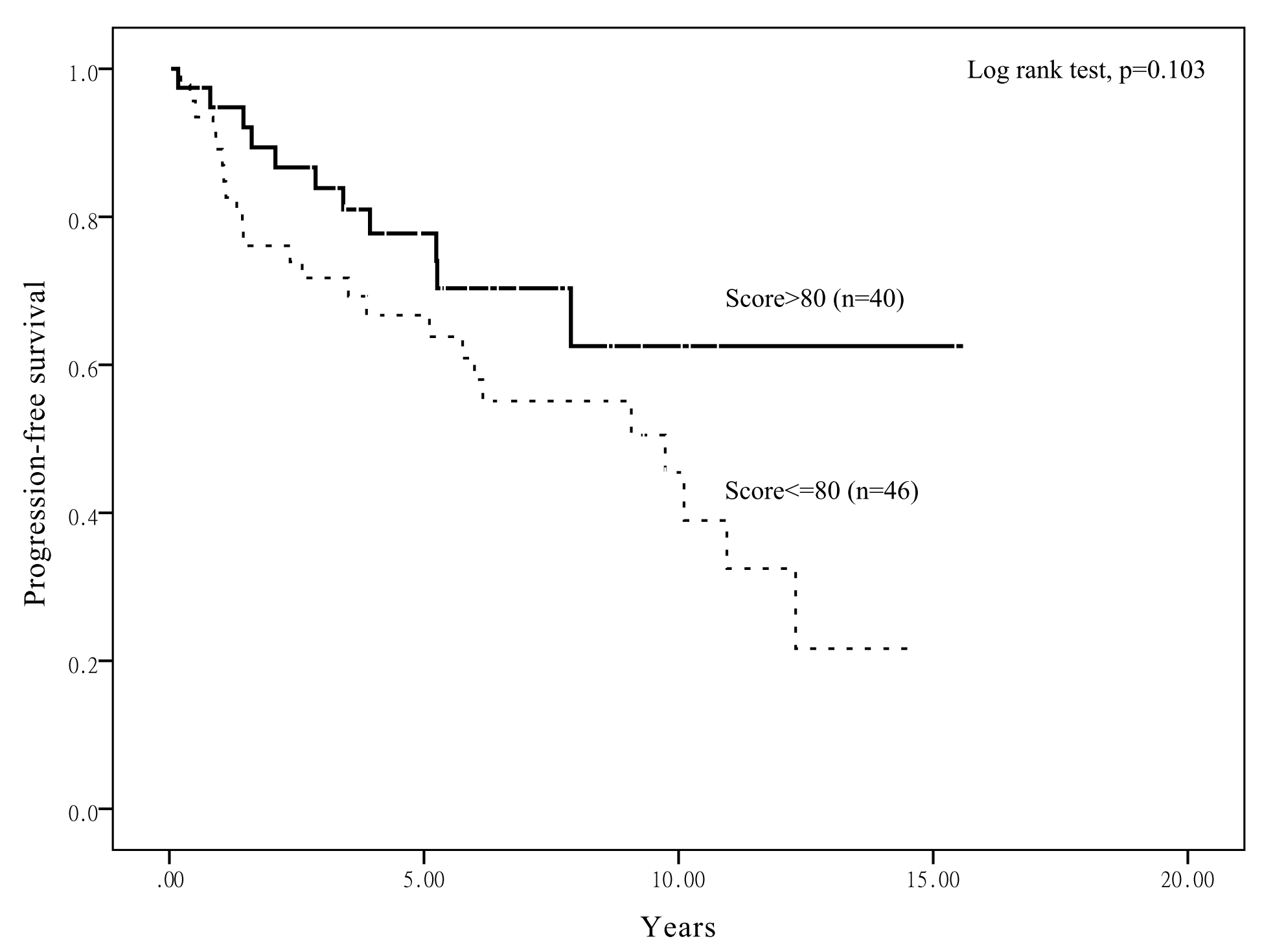

Supplement: S3 Fig — Analysis to assess the prognostic value of the 5-hmC immunoreactivity in relation to PFS. (TIF) [file pone.0259330.s003.tif]

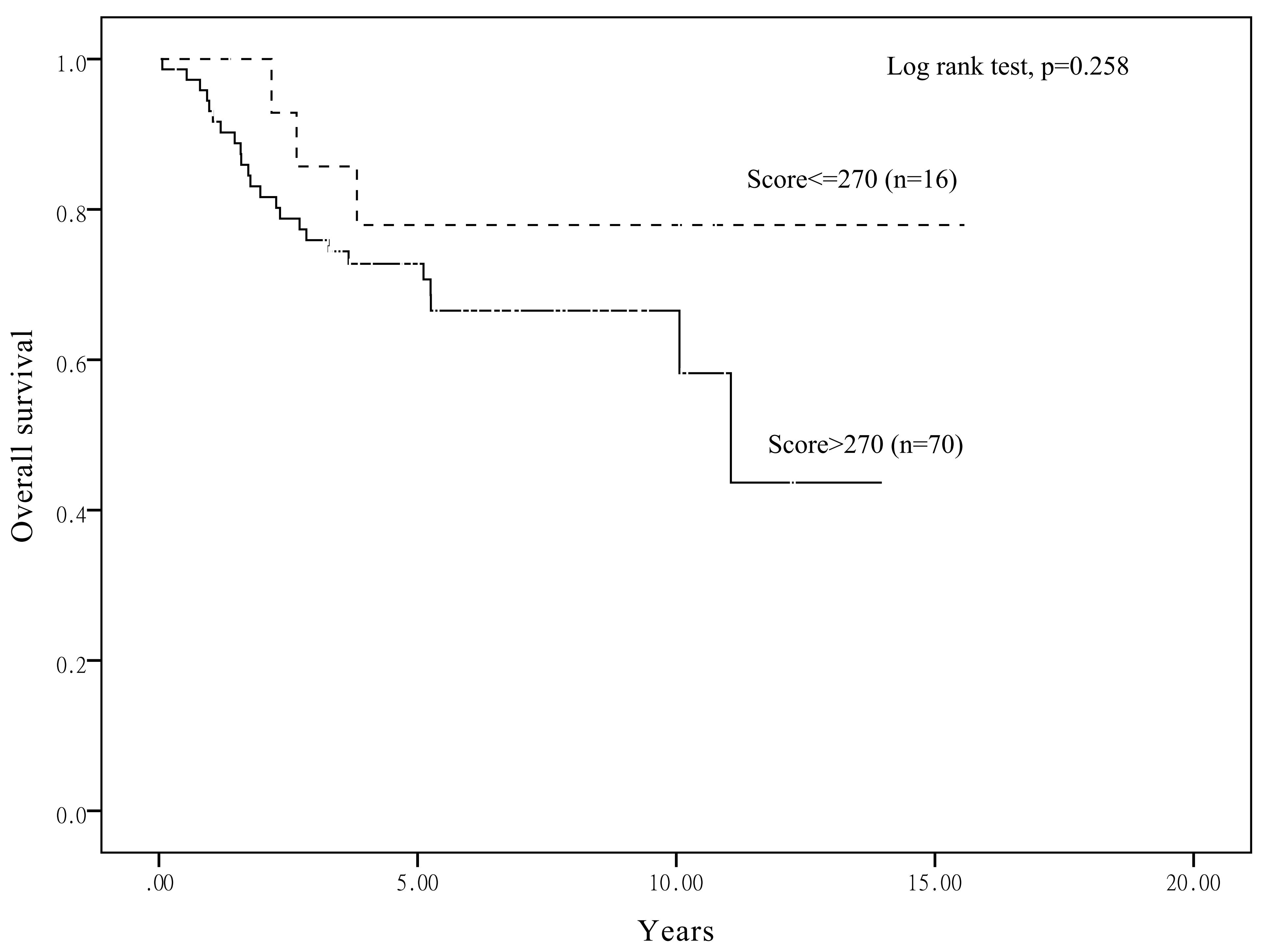

Supplement: S4 Fig — Analysis to assess the prognostic value of the 5-mC immunoreactivity in relation to OS. (TIF) [file pone.0259330.s004.tif]

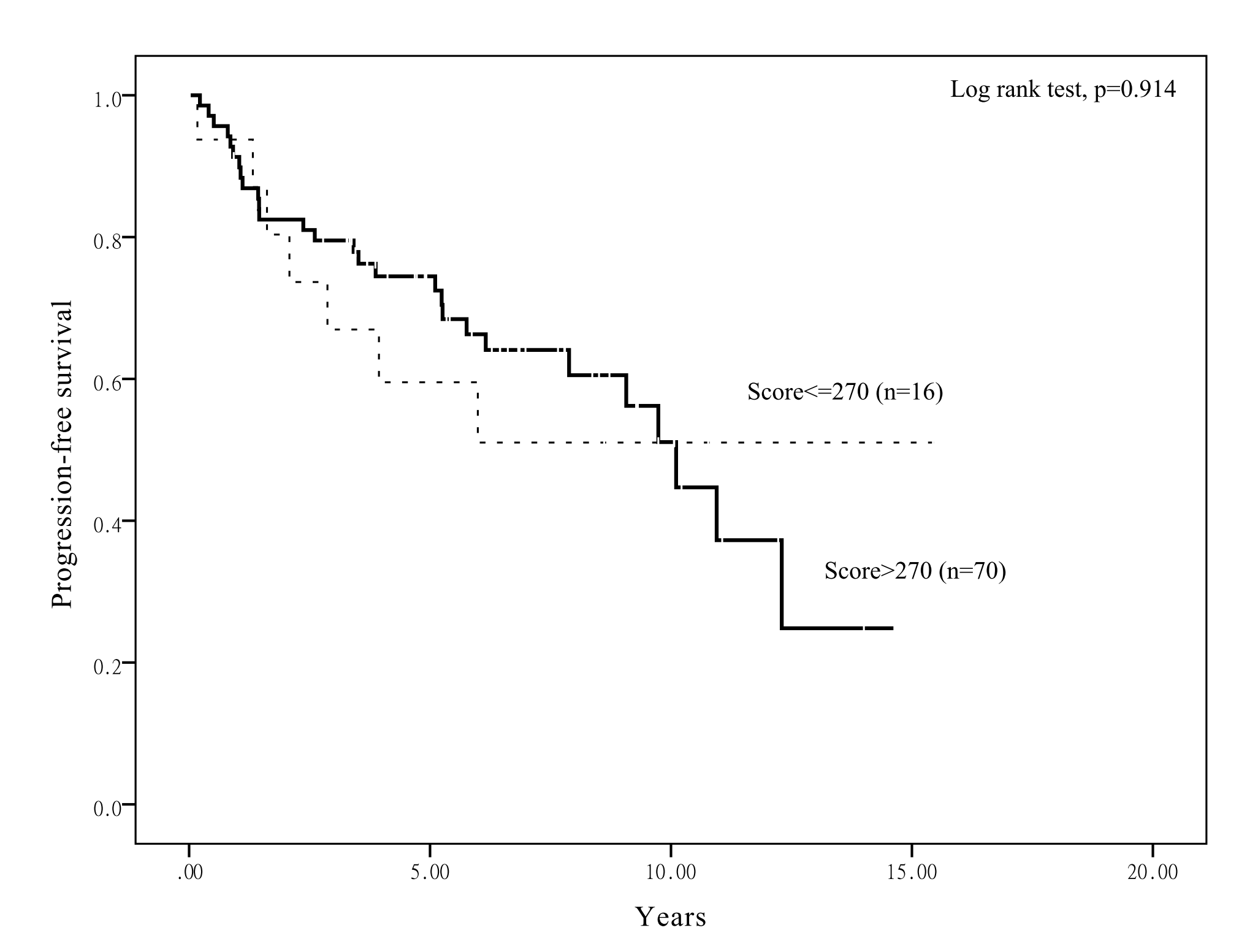

Supplement: S5 Fig — Analysis to assess the prognostic value of the 5-mC immunoreactivity in relation to PFS. (TIF) [file pone.0259330.s005.tif]
